# Purulent pericarditis caused by *Streptococcus pyogenes*: a case report and review

**DOI:** 10.1093/ehjcr/ytaf156

**Published:** 2025-04-03

**Authors:** Vincenzo Somma, Adam Trytell, Andrew MacIsaac

**Affiliations:** Department of Cardiology, St Vincent’s Hospital, 41 Victoria Parade, Melbourne 3065, Australia; Department of Cardiology, St Vincent’s Hospital, 41 Victoria Parade, Melbourne 3065, Australia; Department of Cardiology, St Vincent’s Hospital, 41 Victoria Parade, Melbourne 3065, Australia

**Keywords:** Purulent pericarditis, Endocarditis, Group A Streptococcus, Case Report

## Abstract

**Background:**

Purulent pericarditis is rare and associated with significant mortality. We outline a case of Group A streptococcus (GAS, *Streptococcus pyogenes*) native aortic valve infective endocarditis with associated purulent pericarditis and review the literature. Of the reported cases, most occur in children and have high degrees of mortality. There are no cases that describe GAS purulent pericarditis as a complication of infective endocarditis.

**Case summary:**

A 43-year-old female presented with an altered conscious state and refractory hypotension. An urgent transthoracic echocardiogram revealed a vegetation on the aortic valve, moderate-severe aortic regurgitation and a moderate-large circumferential pericardial effusion with evidence of early cardiac tamponade. An urgent pericardiocentesis was performed and drained 500 mL of purulent pericardial fluid. Peripheral blood and pericardial fluid cultures grew GAS. Unfortunately the pericardiocentesis did not improve the patient’s haemodynamics, she developed recurrent malignant arrhythmias culminating in cardiac arrest and was unable to be resuscitated.

**Discussion:**

To our knowledge this is the first described case of GAS purulent pericarditis arising from infective endocarditis. This case contributes to the limited evidence regarding GAS-related purulent pericarditis. Management includes multidisciplinary care with timely administration of broad-spectrum antibiotics, prompt drainage, and contemplation of early surgical intervention. Our review of the 20 reported cases of GAS purulent pericarditis reveals that only 6 out of 20 patients were over 18 years old and the mortality ranged from 40% to 85%. A high degree of clinical suspicion is required as more than half of the cases are diagnosed at post-mortem.

**Learning points**

- Purulent pericarditis carries a high mortality rate, with figures at 40% even with treatment and escalating to 85% without. Over half of the cases are only diagnosed post-mortem, highlighting the need for strong clinical vigilance.
- Effective management of purulent pericarditis involves the use of broad-spectrum antibiotics, immediate drainage, and consideration of early surgical intervention.

## Introduction

Pericarditis, defined as inflammation of the pericardium, has both infectious and non-infectious aetiologies. While idiopathic and viral pericarditis are the most common forms seen today and are usually benign conditions, purulent pericarditis is rare and associated with significant morbidity and mortality.^1–3^ In the pre-antibiotic era, that majority of patients with purulent pericarditis had an underlying infectious disease.^1^ In contrast, the majority of patients in the post-antibiotic era have underlying non-infectious conditions.^1^

Purulent pericarditis due to Group A Streptococcus (GAS) is rare, with only 20 reported cases in the literature, which includes only six patients 18 years or older. Of the reported cases, there are no described cases of GAS purulent pericarditis as a complication of infective endocarditis. We describe the first case of GAS purulent pericarditis in the context of aortic valve infective endocarditis complicated by at least moderate-severe aortic regurgitation and cardiac tamponade.^4^

## Summary figure

**Table:** 

	Event
**0**	Presentation to hospital with altered conscious state and 7 days of lethargy and worsening dyspnoea. Initial transthoracic echocardiogram revealed aortic valve lesion, moderate-large circumferential pericardial effusion with evidence of early cardiac tamponade. Emergency pericardiocentesis with evidence of right ventricular failure occurring during drainage on concurrent echocardiography. Total 500 mL drained. Admission to ICU following procedure.
**+6 h**	Peripheral blood and pericardial fluid cultures grew Group A streptococcus (GAS, *Streptococcus pyogenes*).
**+8 h**	Unfortunately, pericardiocentesis did not improve the patient’s haemodynamics, then developed recurrent malignant arrhythmias culminating in cardiac arrest and was unable to be resuscitated.

## Case report

A 43-year-old female presented to hospital with an altered conscious state on the background of 2 weeks of lethargy. Her medical history was significant for active intravenous drug use. On arrival she was hypotensive (70/50 mmHg), tachycardic (100 beats/min), hypoxic (3 L oxygen requirement), febrile (temperature >38.0°C) and had an elevated lactate (7 mmol/L, < 2 mmol/L). Initial bloodwork demonstrated marked neutrophilia, with a significantly elevated white cell count of 51.9 × 10⁹/L (reference range: 2–7.5 × 10⁹/L) and platelets at 121 × 10⁹/L. Electrolytes revealed hyponatraemia with a sodium level of 129 mmol/L (135–145 mmol/L), an elevated creatinine of 111 µmol/L, and a raised C-reactive protein of 124 mg/L (<5 mg/L). She was admitted to the intensive care unit with mixed cardiogenic and septic shock requiring intravenous fluid resuscitation with crystalloid and increasing noradrenaline to maintain a satisfactory mean arterial pressure.

**Figure 1:**
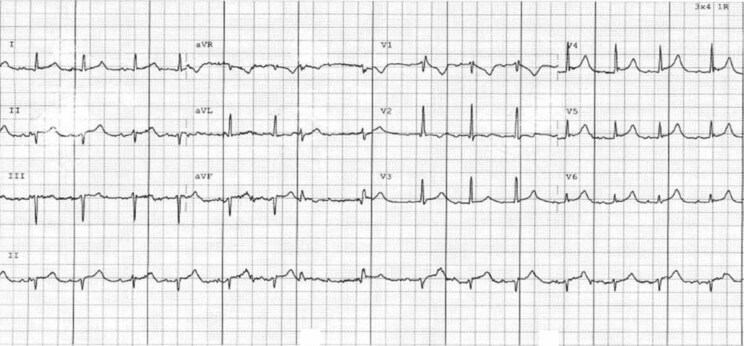
Twelve-lead electrocardiogram at presentation; demonstrating widespread concave ST-segment elevation and slight PR interval prolongation (220 ms).

**Figure 2:**
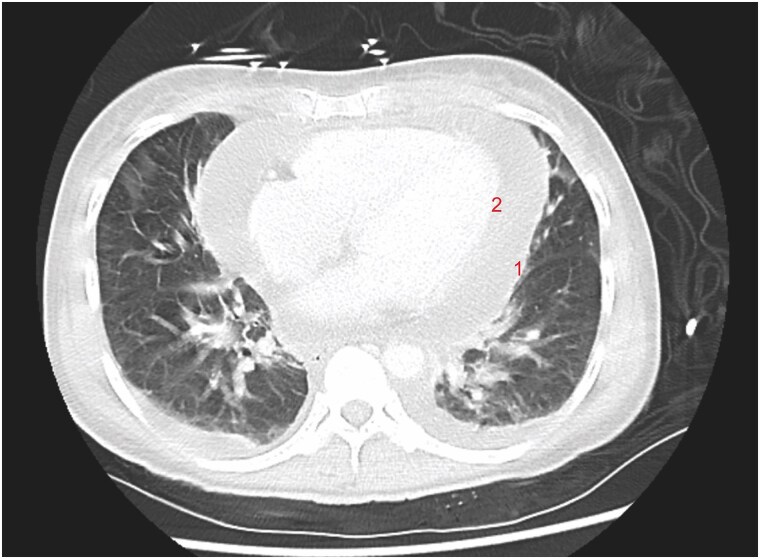
Axial computed tomography scan showing a large circumferential pericardial effusion. The effusion is located between the parietal pericardium (1) and visceral pericardium (2).

**Figure 3:**
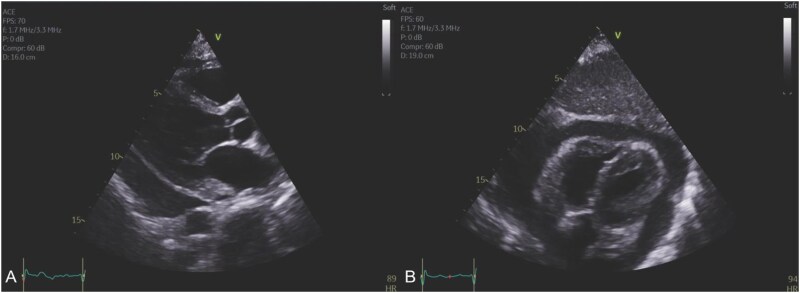
Transthoracic echocardiogram. (*A*) Parasternal long-axis image demonstrating a large pericardial effusion. (*B*) Subcostal image demonstrating early right ventricular diastolic collapse in the context of the large circumferential effusion. Supplementary material online, videos available.

An electrocardiogram revealed widespread ST-segment elevation and slight PR interval prolongation (220 ms) (*Figure 1*). Computed Tomography scan demonstrated a significant pericardial effusion (*Figure 2*) with multiple embolic phenomena to the brain, spleen, and kidney. An urgent transthoracic echocardiogram (TTE) identified a trileaflet aortic valve with a large vegetation that appeared to be attached to the left coronary cusp, a right coronary cusp perforation and at least moderate-severe aortic regurgitation. There was a moderate-large circumferential pericardial effusion with features of heamodynamic compromise including right ventricular diastolic collapse (*Figure 3*). (See Supplementary material online, videos). Blood cultures grew gram-positive cocci, and she was commenced on standard broad-spectrum intravenous antibiotics for infective endocarditis, including ceftriaxone 2 g daily, flucloxacillin 2 g every 4 h, and vancomycin 1.5 g twice daily.

An urgent bedside multidisciplinary discussion occurred between intensive care, cardiology and the cardiothoracic surgical unit to discuss the patient’s emergent management. The consensus was the patient had native aortic valve infective endocarditis complicated by moderate-severe aortic regurgitation with likely seeding into the pericardium resulting in purulent pericarditis with clinical and echocardiographic features of cardiac tamponade. Regardless of the intervention, her mortality risk was extremely high.

Due to haemodynamic instability, uncontrolled sepsis, cerebral septic emboli and an unacceptable risk of intracranial haemorrhagic transformation with cardiothoracic surgery requiring cardiopulmonary bypass, the acute surgical risk of an aortic valve replacement was deemed prohibitive. The patient proceeded to pericardiocentesis to assist with haemodynamic support and for further diagnostic evaluation of the pericardial effusion. The patient was to have ongoing antibiotics and supportive care with the view for surgical aortic valve replacement if the patient responded to initial medical therapy.

**Figure 4:**
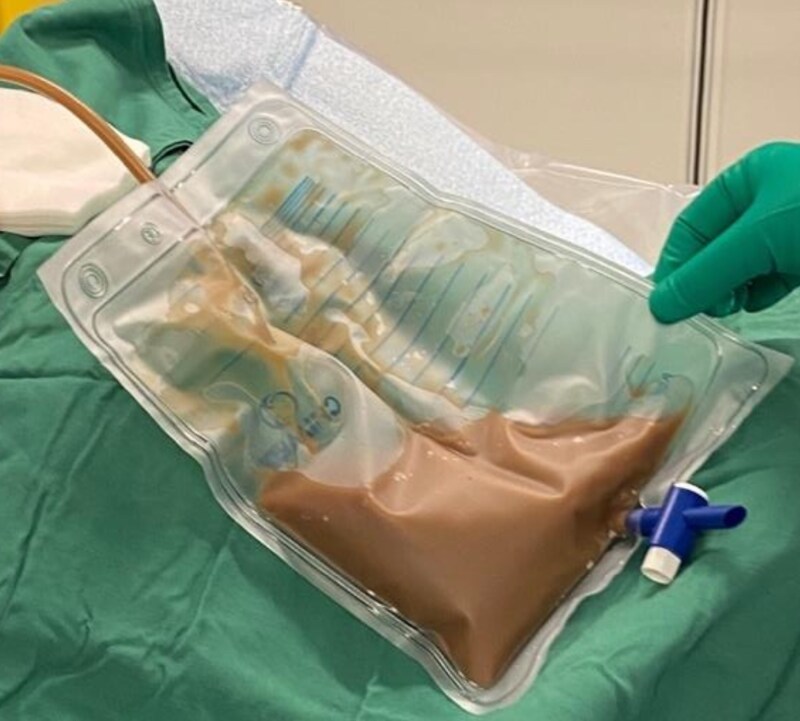
Pericardial aspirate demonstrating purulent pericardial fluid.

The pericardiocentesis was uncomplicated and successfully drained 500 mL of purulent pericardial fluid (*Figure 4*). Complete resolution of the pericardial effusion was confirmed by TTE; however, the patient remained haemodynamically unstable and required increasing ionotropic support. The pericardial fluid grew a large amount of gram-positive cocci, which was later identified as *Streptococcus pyogenes* (GAS). While in ICU, the patient developed ventricular tachycardia followed by prolonged pauses, ultimately resulting in cardiac arrest, and the patient died.

## Discussion

Purulent pericarditis is a rare condition that accounts for <1% of all causes of pericarditis in developed countries and 2% to 3% in developing countries.^5,6^ Purulent pericarditis has a mortality of 40% when treated and 85% if untreated.^7^ Unfortunately, the diagnosis is made post-mortem in more than half the cases. Thus, a high index of clinical suspicion is crucial.^1^ Pericarditis is an uncommon complication of infective endocarditis. Studies estimate its prevalence at ∼8.5%, while autopsy series report higher rates, ranging from 18% to 20%.^8^

The paradigm of purulent pericarditis has changed with the widespread use of antibiotics. Prior to the widespread availability of penicillin, the majority of patients had underlying infectious diseases compared with only 22% in the post-antibiotic era.^1^ The majority of patients in the post-antibiotic era have non-infectious precipitants, including recent thoracic surgery, chronic kidney disease, immunosuppression, alcohol abuse, rheumatoid arthritis or malignancy.^1,9^ In Western countries, the most common organisms isolated from purulent pericardial fluid have been staphylococci, streptococci and pneumococci.^10^ This presentation is the first documented case of GAS purulent pericarditis as a complication of infective endocarditis.

GAS is a gram-positive bacteria that commonly causes mild infections in humans including pharyngitis and impetigo.^11^ However in more severe forms it is termed invasive GAS disease, which is characterized by severe disease including bacteraemia, meningitis and necrotising fasciitis.^12^ Typically invasive GAS infections follow a bi-modal distribution, with patients younger than 5 years or older than 65 years old affected.^12^ Moreover, individuals with a history of intravenous drug use, immunodeficiency, pregnant and post-partum women, homelessness, and those residing in overcrowded housing or lacking access to clean water are susceptible to invasive GAS.^12^ In a large retrospective analysis conducted in Australia from 2007 to 2017, it was found that invasive GAS is uncommon, with a mean annual incidence of 3.1 per 100,000^11^. There have been no reported cases of GAS purulent pericarditis as a complication of infective endocarditis.^13^

Echocardiography plays a crucial role in the diagnosis and management of purulent pericarditis by identifying the pericardial effusion, septations, thickened pericardium, and other associated complications including cardiac tamponade and constrictive pericarditis.^6,14^ Suspicion of purulent pericarditis is an indication for urgent pericardiocentesis, which provides a diagnosis.^14^ Other recommendations include sending peripheral and pericardial fluid for bacterial, fungal and tuberculous culture.^14^

Management of purulent pericarditis requires aggressive management as death is inevitable if untreated.^14^ Empirical intravenous antimicrobial therapy should be commenced until microbiological results are available. Urgent pericardiocentesis is required for diagnostic, as well as therapeutic purposes, in the context of cardiac tamponade.^14^ Purulent pericarditis can often be heavily loculated and rapidly re-accumulate,^15^ thus intrapericardial thrombolysis is a possible treatment to aid achieve adequate drainage^16^ and subxiphoid pericardiostomy and rinsing of the pericardial cavity should be considered.^17^ Despite this, only one previous reported case of GAS purulent pericarditis underwent a pericardial window and no patients received intrapericardial thrombolysis.

Purulent pericarditis is rare, requires a strong index of suspicion and carries a high mortality rate. This is the first reported case of GAS purulent pericarditis as a complication of infective endocarditis. This case adds to the limited body of evidence regarding GAS purulent pericarditis and reminds clinicians to manage purulent pericarditis aggressively with broad-spectrum antibiotics, prompt drainage and to consider early surgery.

## Lead author biography


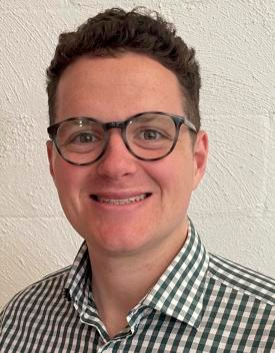


Vincenzo currently works at St Vincent's Hospital Melbourne and has an interest in Cardiology.

## Supplementary Material

ytaf156_Supplementary_Data

## Data Availability

Deidentified data are available upon reasonable request.

## Notes

**Consent:** The authors confirm that written consent for submission and publication of this case report has been obtained from the patient in line with the Committee on Publication Ethics guidance.

**Funding:** None declared.
